# Striae Distensae: Clinical Results and Evidence-Based Evaluation of a Novel 675 nm Laser Wavelength

**DOI:** 10.3390/medicina59050841

**Published:** 2023-04-26

**Authors:** Alice Verdelli, Paolo Bonan, Irene Fusco, Francesca Madeddu, Domenico Piccolo

**Affiliations:** 1 Laser Cutaneous Cosmetic and Plastic Surgery Unit, Villa Donatello Clinic, 50121 Florence, Italy; 2 El.En. Group, 50041 Calenzano, Italy; 3 Skin Centers, 67051 Avezzano, Italy

**Keywords:** Manchester Scar Scale, 675 nm laser, striae distensae

## Abstract

*Background*: A current popular aesthetic problem, especially among younger women, is striae distensae (SD), also referred to as “stretch marks.”. *Aim*: The potential use of the 675 nm laser has been investigated in the treatment of SD. *Methods*: Patients underwent three sessions of the 675 nm laser with a 1-month interval between sessions. A total of three sessions were performed. The Manchester Scar Scale was used to assess stretch mark changes, and the mean scores related to each parameter at baseline and 6M FU after the last treatment session were measured. A clinical photographic evaluation was performed to show the aesthetic improvement of SD. *Results*: The patients’ treated areas were the abdomen, thighs, buttocks, and breasts. Mean scores related to each Manchester Scar Scale parameter, with their relative percentage change, at baseline and 6M FU after the last treatment session were significantly improved. The total mean Manchester Scar Scale score significantly diminished from 14.16 (±1.30) to 10.06 (±1.32) at 6M FU (*p* < 0.01). The clinical photographs showed promising aesthetic SD improvement. *Conclusions*: 675 nm laser therapy demonstrated a good tolerance for the treatment of stretch marks applied to various body areas preventing any discomfort for the patient and with a significant improvement in skin texture.

## 1. Introduction

Striae distensae (SD), also known as “stretch marks”, are a common aesthetic problem, especially in young women. Clinically characterized by atrophic linear lesions, first reddish, then porcelain-white, they are mainly localized on the abdomen, breasts, and thighs in adolescents, they are common on the thighs, buttocks, breasts (females), and back (males) [1]. SDs generally appear during puberty, pregnancy, and in case of rapid change in weight (loss or gain). They can also be found in association with endocrinological diseases such as Cushing’s syndrome or genetic pathologies such as Marfan syndrome and especially after long-standing therapies with local or systemic corticosteroids or other drugs (chemotherapy, prolonged antibiotic therapy, contraceptives, neuroleptics, and anti-retroviral protease inhibitors (indinavir)) [2].

Genetic predisposition in association with hormonal changes, drugs, and mechanical stress contribute to SD occurrence, but the exact pathogenesis remains unclear. The main hypothesis is structural damage of dermal collagen and elastic tissue. In fact, an alteration of the architecture of the dermal connective tissue, with the involvement of the extracellular matrix (ECM), has been demonstrated along with damage to fibrillin, elastin, fibronectin, and collagen fibers. Elastases released from mast cells and macrophages seem to be responsible for the initial structural dermal alterations followed by a reorganization of collagen and fibrillin. The degradation of collagen finally results in atrophic scarring.

Recently, the 675 nm wavelength showed successful results in facial aging [3,4,5] as well as for remodeling atrophic scars in patients with acne [6] and melasma management, [7] even in dark phototypes [8].

A proven effect of this laser system has been reported also for facial rejuvenation in Asian population [9]. From this perspective, the 675 nm laser could have a potential use also in the treatment of SDs. On these bases, this work investigates the findings of a prospective, pilot study on the efficacy and safety of 675 nm laser on SD management.

## 2. Materials and Methods

### 2.1. Patients Populations and Study Device

This study’s goal was to assess the clinical effectiveness of a diode laser with a wavelength of 675 nm (RedTouch laser, DEKA M.E.L.A., Calenzano, Italy) for the non-invasive treatment of stretch marks (Striae distensae) reduction on the patient’s abdomen, thighs, buttocks, and breasts. Specifically, a total of 6 abdomens, 6 thighs, 7 buttocks and 13 breasts were treated. The laser device is a continuous (CW) diode laser that emits a wavelength of 675 nm, and it is equipped with a 13 × 13 mm scanning system, which can be accessorized with contact and temperature sensors integrated into the handpiece and is able to generate fractional micro-zones (DOT) of 0.7 mm width of either sub-ablative and selective thermal damage on the skin. Each DOT receives radiation from the CW source via the Power and Dwell time parameters. By adding a distance (Spacing) between each DOT, the scanning system enables frac-tionally uniformly covering the treatment area. This preserves the epidermal layer thanks to an integrated skin cooling system, protecting skin health while minimizing downtime. Each emission may reach a thermal column depth of more than 1 mm. Before starting the study, informed consent was obtained from all the participants. A total of 32 female patients with SD, ranging in Fitzpatrick skin phototype from I to IV (5 patients present phototype I, 13 patients present phototype II, 8 patients present phototype III, 6 patients present phototype IV) and with a mean age of 31 (±7.5), were enrolled. Study exclusion criteria included: (1) hypersensitivity to light in the red and near-infrared wavelength region; (2) use of photosensitizing drugs, anticoagulants, and/or immunosuppressants; (3) photosensitivity diseases; (4) pregnancy; (5) personal or family history of skin cancer, (6) sun exposure in the previous month; and (7) the presence of tattoos or skin disorders on the areas to be treated.

### 2.2. Study Protocol

Patients were treated with 3 sessions of the 675 nm laser using a conductive gel (power 5–10 W, dwell time 100–200 ms, spacing 500–1500 µm and stack 1) with a 1-month interval between sessions. Areas to be treated were cleaned with a mild soap, rinsed with water, and topical anesthetic was applied before the session started. Based on the subject’s skin type and level of tolerance, the energy treatment assessment was performed for each patient on a specific area “test.”. Within 48–72 h, the test’s outcome was noticeable. Mild erythema was recognized as the endpoint. Treatment was administered by softly passing the handpiece over the skin’s surface, within spots close to each other without overlapping but without leaving untreated areas. Following treatment gauzes soaked in cold water were used to cool the skin.

### 2.3. Outcomes Assessment

The Manchester Scar Scale [10] was used to assess stretch mark changes, the mean scores related to each parameter at baseline, and at 6 months follow-up (M FU) after the last treatment session, were measured. The Scale’s parameters are five: contour, color, texture, distortion, and finish. All parameters range in a score scale of 1 to 4, except for the finish, which was scored either 1 (matte) or 2 (shiny). These scores can range from 18 points at the highest to 5 points at the lowest (lower scores indicate a better aesthetic appearance). A clinical photographic evaluation was performed to show the aesthetic SD improvement.

### 2.4. Side Effects

Adverse events such as edema, burns, formation of keloids, excessive pain, crusting, scars, prolonged erythema, blisters, or dyschromia were evaluated at the post-treatment visit.

### 2.5. Statistics

Paired Student’s *t*-test was used to test all the outcome data for statistical significance with the SPSS program version 25.0 (IBM, Armonk, NY, USA). The level of significance for statistical tests was (*p* < 0.01). Data were represented as means ± standard deviation (SD).

## 3. Results

**Figure 1:**
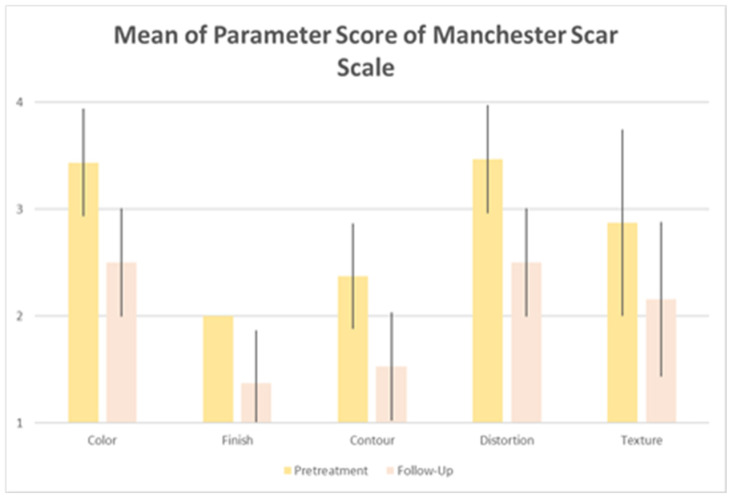
Histogram of patient’s mean of each parameter score of the Manchester Scar Scale.

**Figure 2:**
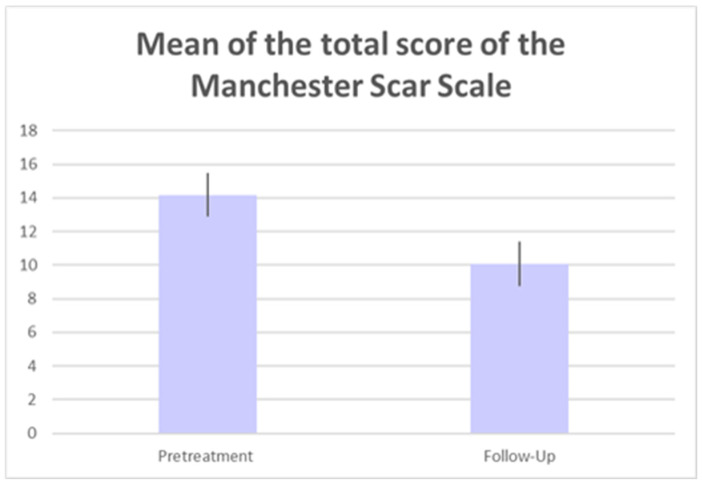
Histogram of patient’s total mean score of the Manchester Scar Scale.

**Figure 3:**
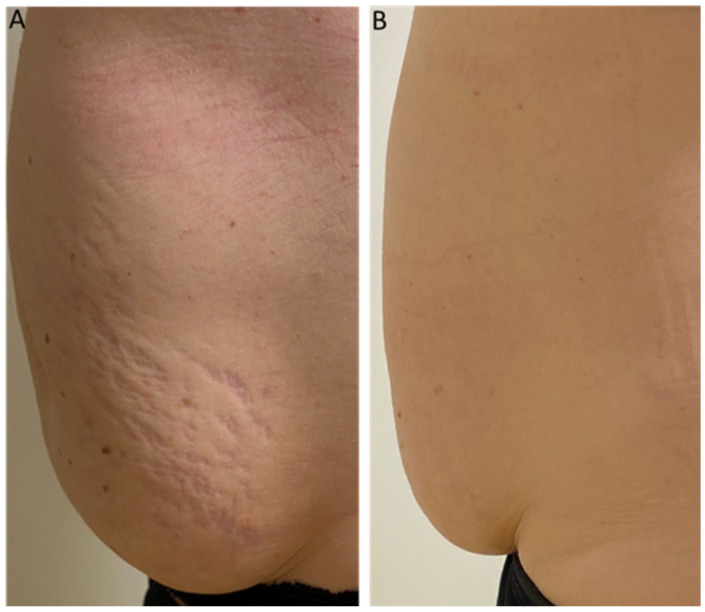
Analysis of 2D photographs of patient’s striae affecting abdominal zone from baseline (**A**) to 6MU FU (**B**) revealed an average reduction in the striae volume, texture, redness and color following 675 nm laser treatment.

**Figure 4:**
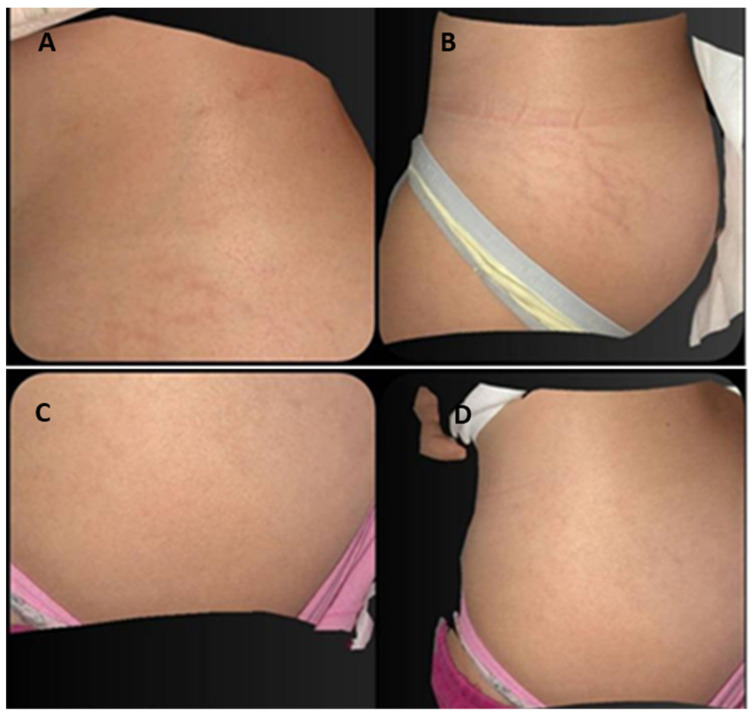
Pre-treatment (**A**,**B**) and post-treatment (**C**,**D**) of 2D Images analysis of the same patient’s buttocks treated zones. An overall reduction in the color, redness, volume, and texture of striae after three treatments with 675 nm laser is observed.

The patient’s treated areas were the abdomen, thighs, buttocks, and breasts. Mean scores related to each Manchester Scar Scale parameter, at baseline and 6 M FU after the last treatment session significantly improved (*p* < 0.01) showing excellent results as reported in Table 1 and Figure 1. Color significantly reduced from baseline 3.44 (±0.50) to 2.50 (±0.50) at 6M FU. Finish was significantly lower from baseline 2.00 (±0.00) to 1.37 (±0.50) at 6M FU, as well as the contour from baseline 2.37 (±0.50) to 1.53 (±0.50) at 6M FU. Additionally, distortion and texture lowered from baseline 3.47 (±0.50) to 2.5 (±0.50) at 6M FU and from baseline 2.87 (±0.87) to 2.16 (±0.72) at 6M FU, respectively. The total mean Manchester Scar Scale score significantly diminished from 14.16 (±1.30) to 10.06 (±1.32) at 6M FU (*p* < 0.01) (Figure 2). The clinical photographs showed promising aesthetic improvement of SDs, as shown in Figure 3 and Figure 4. None of the monitored side effects were detected except for mild erythema, which normally represents an endpoint of the treatment which resolves within 24 h.

## 4. Discussion

**Figure 5:**
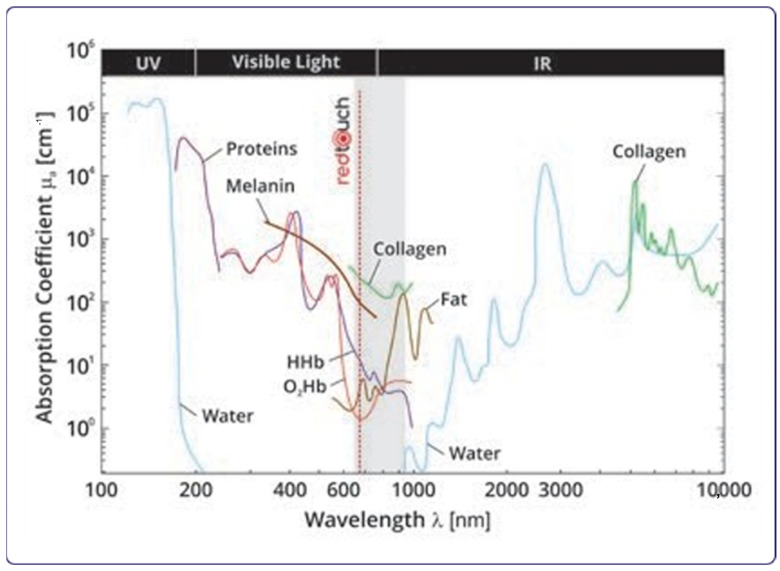
Absorption spectrum of the various chromophores in human tissue. An optical window is observed and includes the wavelength of 675 nm, with high absorption by the concomitant collagen. Courtesy of DEKA MELA.

Stretch marks’ treatment is still difficult even though several therapeutic options are currently available. Accordingly, the available treatments for SD work mainly on collagen synthesis, but despite a multitude of options, no single treatment has yet proven effective [11]. They include topical agents, chemical peels, microdermabrasion, and ablative and non-ablative energy devices [12,13,14,15,16,17,18]. Currently, the most used treatment for SDs are fractional lasers and Pulsed dye lasers (PDL) [19,20,21,22]. For the treatment of SD, a number of ablative or non-ablative laser strategies are employed, as well as photodynamic therapy [23,24,25]. Specifically, lasers with wavelengths between 524 and 904 nm have been demonstrated to speed up the wound-healing process, boost collagen synthesis, enhance epithelial differentiation, and stimulate dermal vascularity in many wound models [26,27,28]. Different laser parameters have been tested for SD treatment, either alone or in conjunction with other treatment modalities. In our research, three treatment sessions with the non-invasive 675 nm laser led to a positive effect on SD management. This non-ablative laser system emits a 675 nm wavelength through a 13 × 13 mm scanning system capable of generating sub-ablative microzones on the skin with selective thermal damage, reaching a depth of more than 1 mm, inducing minimal thermal effects. Based on its spectral absorbance, this new device with a wavelength of 675 nm acts directly on the collagen component [4], and the heat is transferred directly to the collagen fibers without hitting other chromophores (see Figure 5). The selectivity of its emission allows it to act within an optical window that maximizes the affinity with collagen fibers and minimizes interactions with the vascular component. This mechanism of action translates into a procedure that uses minimal energy levels that facilitate the execution of the treatment and does not require special preparations of the skin at the same time.

The energy delivered to the collagen can induce regeneration, promoting the production of dermal collagen, and the straightening of elastic fibers. Indeed, the newly created thermal column diffuses heat to the surrounding areas causing immediate shrinkage and denaturation of the collagen with subsequent new collagen formation through the stimulation of fibroblasts and neocollagenesis activation.

This diode laser with a wavelength of 675 nm generates 1 mm microzones of thermal damage that, thanks to the cooling and selectivity of the dermal layer, do not damage the epidermal layer. Thanks to the increased focus on small spots, microscopic necrotic epidermal debris or dermo-epidermal detachment, which is typical of the post-operative course of Near Infrared (NIR) systems, were not observed. Our study findings pointed to a notable SD improvement for all patients, as well as a significant improvement in finish, texture, color, distortion, and contour, along with a significant increase in skin elasticity as clearly shown in the photographic evaluation. Due to the preventive cooling of the skin, the procedure is also relatively painless. The treatment is simple to administer, non-invasive and did not have reported side effects or complications.

### Limitations of the Study

Limitations include the limited number of patients and the lack of a histologic investigation.

## 5. Conclusions

The efficacy of 675 nm laser for treating stretch marks on the abdomen, thighs, buttocks, and breasts showed good tolerance with a significant improvement in skin texture and quality of stretch marks, and temporarily and minimally restricts the patient’s daily activities preventing any discomfort for the patient.

## Figures and Tables

**Table 1:** Mean parameter scores of Manchester Scar calculated at baseline and 6M FU after the last laser treatment session.

Parameters of Manchester Scar Scale *N* = 32	Baseline Mean ± SD	6M FU Mean ± SD	(Significance)
Colour (Score range, 1–4)	3.44 ± 0.50	2.50 ± 0.50	(*p* ˂ 0.01)
Finish (Score range, 1–2)	2.00 ± 0.00	1.37 ± 0.50	(*p* < 0.01)
Contour (Score range, 1–4)	2.37 ± 0.50	1.53 ± 0.50	(*p* < 0.01)
Distortion (Score range, 1–4)	3.47 ± 0.50	2.5 ± 0.50	(*p* < 0.01)
Texture (Score range, 1–4)	2.87 ± 0.87	2.16 ± 0.72	(*p* < 0.01)
Total score	14.16 ± 1.30	10.06 ± 1.32	(*p* < 0.01)

## Data Availability

Data supporting this study’s findings are available on request from the corresponding author (IF).
